# Diversity of Protein and mRNA Forms of Mammalian Methionine Sulfoxide Reductase B1 Due to Intronization and Protein Processing

**DOI:** 10.1371/journal.pone.0011497

**Published:** 2010-07-09

**Authors:** Xinwen Liang, Dmitri E. Fomenko, Deame Hua, Alaattin Kaya, Vadim N. Gladyshev

**Affiliations:** 1 Department of Biochemistry and Redox Biology Center, University of Nebraska-Lincoln, Lincoln, Nebraska, United States of America; 2 Division of Genetics, Brigham and Women's Hospital and Harvard Medical School, Boston, Massachusetts, United States of America; The University of Akron, United States of America

## Abstract

**Background:**

Methionine sulfoxide reductases (Msrs) are repair enzymes that protect proteins from oxidative stress by catalyzing stereospecific reduction of oxidized methionine residues. MsrB1 is a selenocysteine-containing cytosolic/nuclear Msr with high expression in liver and kidney.

**Principal Findings:**

Here, we identified differences in MsrB1 gene structure among mammals. Human MsrB1 gene consists of four, whereas the corresponding mouse gene of five exons, due to occurrence of an additional intron that flanks the stop signal and covers a large part of the 3′-UTR. This intron evolved in a subset of rodents through intronization of exonic sequences, whereas the human gene structure represents the ancestral form. In mice, both splice forms were detected in liver, kidney, brain and heart with the five-exon form being the major form. We found that both mRNA forms were translated and supported efficient selenocysteine insertion into MsrB1. In addition, MsrB1 occurs in two protein forms that migrate as 14 and 5 kDa proteins. We found that each mRNA splice form generated both protein forms. The abundance of the 5 kDa form was not influenced by protease inhibitors, replacement of selenocysteine in the active site or mutation of amino acids in the cleavage site. However, mutation of cysteines that coordinate a structural zinc decreased the levels of 5 and 14 kDa forms, suggesting importance of protein structure for biosynthesis and/stability of these forms.

**Conclusions:**

This study characterized unexpected diversity of protein and mRNA forms of mammalian selenoprotein MsrB1.

## Introduction

Oxidation of proteins during aging or under conditions of oxidative stress may lead to alterations in protein structure and function and has been linked to age-related diseases [1], [2]. Methionine sulfoxide reductases (Msrs) are repair enzymes, which reduce oxidized methionine residues in proteins in a stereospecific manner. Two such protein families are known, including MsrA and MsrB that act on methionine S-sulfoxide (Met-SO) and methionine R-sulfoxide (Met-RO), respectively. Mammals contain three MsrB isozymes localized to different cell compartments. One of them is MsrB1 (previously known as SelR or SelX) that localizes to the nucleus and cytosol. It is a selenoprotein that contains selenocysteine (Sec) in place of cysteine normally present in the catalytic sites of MsrBs and MsrAs [3].

The role of MsrA in protecting cells against oxidative stress has been well documented in bacteria, yeasts, plants and mammals [4], [5], [6], [7], [8], [9], [10], [11]. There are also many reports of this function for MsrB [9], [11], [12], [13], [14], [15], [16]. Under certain conditions, overexpression of MsrB can increase lifespan in yeast [12]. Overexpression of MsrB2 protects MOLT-4 cells against oxidative stress [9], [14]. The enzymatic activities of MsrA and MsrB were significantly lower in the organs of aged animals [11]. Our previous studies showed increased levels of malondialdehyde, protein carbonyls, protein methionine sulfoxide, and oxidized glutathione in liver and kidney of MsrB1 knockout mice [13]. In addition, two protein forms (14 and 5 kDa) of MsrB1 were detected in mouse tissues. Mass spectrometry analysis of the 5 kDa protein suggested that this short form corresponds to the C-terminal fragment of MsrB1 starting with Asn76 [13]. However, the mechanism of formation and biological functions of this form are not known.

In the present study, we analyzed recombinant mouse MsrB1 and its Sec-to-Cys mutant protein expressed in HEK 293 cells and *E. coli*. Our research suggests that the 5 kDa form was not produced due to alternative translation initiation and does not require Sec insertion, but that its levels are regulated by the stability of MsrB1 structure.

The 3′-untranslated regions (UTRs) often contain regulatory elements that govern spatial and temporal properties of mRNAs, including mRNA stability, localization and translation [17]. About half of mammalian genes use alternative polyadenylation to generate multiple mRNA isoforms [18], [19], [20]. Our analysis of MsrB1 cDNA sequences revealed two forms of the 3′-UTR in mice and a single form in humans. Further analyses identified differences in gene structure between rodents and most other mammals, including humans, due to the process of intronization wherein exonic sequences were recruited to generate a new intron within the 3′-UTR. We report characterization of these mRNA and protein forms of MsrB1.

## Materials and Methods

### Computational analyses of MsrB1 sequences

Sec-containing MsrB1 sequences were collected using TBLASTN. Mouse MsrB1 protein sequence was used as a query sequence to search against NCBI EST database. Sequences containing Sec encoded by TGA codon were analyzed for occurrence of SECIS elements using SECISearch. EST sequences with predicted SECIS elements were searched against NCBI genome and non-redundant nucleotide databases to assess genomic sequences and against NCBI EST database to examine splicing and alternative splicing events. All selected sequences were analyzed for splice donor and acceptor sites with NetGene2 Server. Only *Mus musculus* and *Rattus norvegicus* genomic DNA possessed reliable splice sites within the 3′-UTR. MsrB1 structure was modeled with Modeler 8.2.

### Quantification of MsrB1 mRNA expression in C57BL/6 mice

Total RNA was extracted from previously frozen various tissues of two C57BL/6 mice with RNAqueous®Kit (Applied Biosystems/Ambion, Austin, TX). Purified RNA samples (1 µg) were treated with RNAse-free DNAse (Fermentas), and then used to synthesize cDNA using M-MulV reverse transcriptase and Oligo dT primers (Fermentas) according to manufacturer's instructions. The primers for the analysis for MsrB1 mRNA expression were designed by GenScript Real Time Primer Design Program and they are as follows: forward, 5′-CTTCGGAGGCGAGGTTTTCC-3′, and reverse, 5′-TCTCAGGGCACTTGGTCACA-3′, which produce a 164 bp amplicon corresponding to the MsrB1 coding region between exons 1 and 2. Primers specific for the MsrB1 Form 2: forward, 5′-TGGAAGGTTTCCACTGCTCT-3′, and reverse, 5′- AAGGAGATTGGTGGCAGTTT -3′, generate a 126 bp amplicon. GAPDH expression was used as control and the primers were 5′-TCACCACCATGGAGAAGGC-3′ (forward) and 5′-GCTAAGCAGTTGGTGGTGCA-3′ (reverse) [21]. The PCR amplification mixture (25 µl) contained 25 ng template cDNA, 12.5 µl 2X SYBR green I master mix buffer (Bio-Rad), and 300 nM forward and reverse primers. Each assay (in triplicate) included a non-template control and a non-RT control. Reactions were run on a MyiQ real-time PCR detection system (Bio-Rad). Gene expression was analyzed by Gene Expression Macro (Bio-Rad) and PCR products were also analyzed by 2% agarose gel electrophoresis to confirm product size and specificity.

### Cloning, expression, purification, detection of recombinant mouse MsrB1

To express MsrB1 in mammalian cells, the following constructs were generated: wild-type and cysteine (Cys) mutant cDNA of mouse MsrB1 (NM_013759) corresponding to Form 1 3′-UTR were amplified from pET28a (+) MsrB1-Sec and pET28a (+) MsrB1-Cys as templates that were previously constructed in the laboratory. The cDNA for MsrB1 corresponding to Form 2 3′-UTR was amplified from a mouse EST clone (IMAGE: 6432555, Open Biosystems, Huntsville, AL). The PCR products were sequence verified and cloned into Xho1/EcoR1 sites of pEGFP-C3 to generate constructs coding for proteins with N-terminal GFP tags. The resulting constructs were named as pEGFP-MsrB1-F1 (with Form 1 3′-UTR), pEGFP-MsrB1-U95C (with Form 1 3′-UTR), and pEGPC3-MsrB1-F2 (with Form 2 3′-UTR). All other mutant forms of MsrB1 were generated by site-directed mutagenesis using Stratagene's QuickChange site-directed mutagenesis kit and pEGFP-MsrB1-F1 as a parent DNA.

To express wild-type and mutant forms of GFP-tagged MsrB1 in HEK 293 cells, constructs were transfected into cells using a calcium phosphate method. The transfected cells were cultured in DMEM medium supplemented with 10% newborn bovine serum for 48 h at 37°C in the atmosphere of 5% CO2. To extract the protein, cell pellets were lysed with mammalian cell lysis reagent (Sigma) containing complete protease inhibitor mixture (Roche) for 15 min at room temperature, cell lysates were spun down for 10 min at 4°C, and 30 µg of soluble protein was resolved by SDS-PAGE and transferred onto polyvinylidene difluoride membranes. MsrB1 was detected by immunoblot assays with polyclonal antibodies against mouse MsrB1 or visualized by PhosphorImager if cells were metabolically labeled with ^75^Se.

The construct pET28a(+) MsrB1-Cys, described previously [22] was used for expression of MsrB1-Cys mutant containing an N-terminal His-tag in *E. coli* BL21 (DE3). Based on the above construct, a factor Xa cleavage site (IEGR) was inserted into the MsrB1 coding region between Gly75 and Asn76, a previously predicted cleavage site in MsrB1 [13]. Recombinant proteins were expressed in *E. coli* by inducing protein expression in log phase cultures with 0.2 mM isopropyl-D-1-thiogalactopyranoside (IPTG) at 30°C for 4 h, and proteins were purified using cobalt-conjugated affinity resins (TALON Metal Affinity Resin, Clontech, Mountain View, CA) according to the manufacturer's instructions.

### Metabolic labeling of cells with ^75^Se

[^75^Se]-Selenite (specific activity 1,000 Ci/mmol) was obtained from the Research Reactor Facility, University of Missouri (Columbia, MO). HEK 293 cells transfected with the constructs were maintained for 24 h and then metabolically labeled with ^75^Se for 16–24 h as described [23]. Extracts from these cells were subjected to SDS-PAGE, proteins were transferred onto polyvinylidene difluoride membranes, and selenoproteins were detected with a PhosphorImager.

### MsrB activity assays

MsrB1 activity was measured using DTT as a reductant. 100 µl reaction mixtures contained 20 mM DTT, 200 µM dabsyl-Met-RO, and 0.5–20 µg protein in PBS. The reaction was carried out at 37°C for 30 min and products were analyzed by an HPLC method as described previously [24].

### Extraction of proteins from mouse tissues

Mouse tissues were homogenized on ice in PBS, pH 7.4, containing complete protease inhibitor, EDTA and PMSF, centrifuged and the supernatants were used for activity and immunoblot assays.

## Results

### Identification of two forms of mouse MsrB1 mRNA

**Figure 1:**
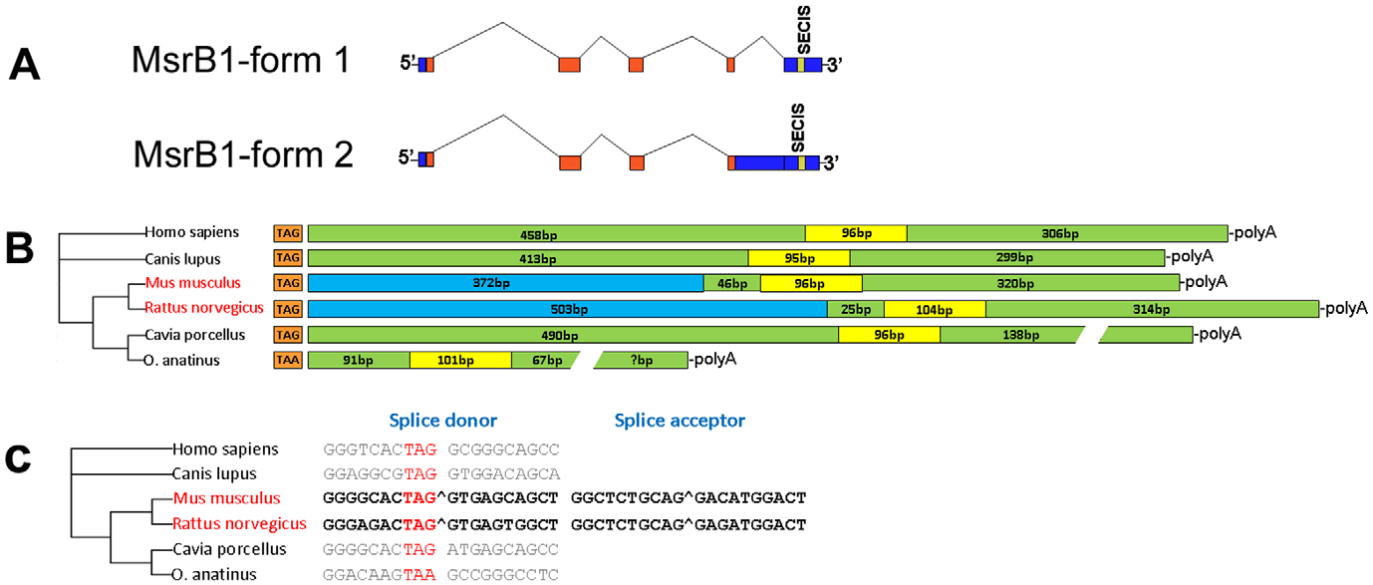
Alternative splice forms of MsrB1. (A) Genomic structure of mouse MsrB1. Red boxes indicate exons corresponding to the coding region, blue boxes show 3′-UTRs, and yellow boxes show the SECIS element. Top panel shows the Form 1 MsrB1 mRNA which lacked intron 4 due to the process of intronization. Bottom panel represents the ancestral form, Form 2 MsrB1 mRNA. (B) Schematic representation of 3′-UTRs of mammalian MsrB1 sequences. The stop codon is shown in orange, 3′-UTRs in green, alternatively spliced regions in blue, and SECIS elements in yellow. (C) Splice donor and acceptor sites in the 3′-UTR of MsrB1. The stop codon is shown in red. Intron-exon junctions are indicated by “∧”.

Computational analysis of mammalian MsrB1 ESTs revealed two cDNA forms in mice that differed in the 3′-UTR. One cDNA form, further designated as Form 1, had 462 bp in its 3′-UTR, and the other, Form 2, 834 bp. The two forms yielded identical predicted ORFs. Compared to Form 1, Form 2 had an extra 372 nucleotides at the 5′ end of the 3′-UTR. Aligning these two MsrB1 mRNA sequences to the mouse genome sequence revealed the presence of an extra intron (intron 4), located immediately following the stop codon, i.e., Form 1 lacked a larger part of the 3′-UTR that was present in Form 2 (Fig. 1A).

### Rodent-specific intronization to generate two mRNA forms

Searches for homologs of mouse Forms 1 and 2 in NCBI dbEST database found matches to rat (*Rattus norvegicus*), guinea pig (*Cavia porcellus*), platypus (*Ornithorhynchus anatinus*), and human (*Homo sapiens*) sequences. Interestingly, all rat sequences corresponded to mouse Form 1 MsrB1 cDNA, whereas all other organisms were represented by Form 2 sequences (Fig. 1B). Further analysis revealed a splice donor site immediately following the stop codon and a splice acceptor site about 400 nucleotides downstream in mouse and rat sequences, whereas these sites were ambiguous or undetectable in other organisms (Fig. 1C). Thus, there were two alternative mRNA forms of MsrB1 in mammals, but both of these forms were only present in mice. Other mammals had single forms, either Form 1 or Form 2. The ancestral form was apparently Form 2 because it was present in diverse mammals, whereas Form 1 was in a small subset of closely related rodents. Thus, in some rodents (i.e., mice and rats, but not guinea pigs), an additional intron evolved within the 3′-UTR through the process known as intronization, i.e., recruitment of exonic sequences to generate an intron. Interestingly, this intron did not include the SECIS element, which is essential for Sec insertion into MsrB1 and located in the region of the 3′-UTR that was shared by Form 1 and Form 2.

### Alternative splicing in mouse MsrB1

As mentioned in the previous section, mouse was the only mammal detected to possess two forms, wherein it occupied an intermediate position between human and other non-rodent mammals (Form 2) on one hand and rat (Form 1) on the other. To further examine the significance of alternative splicing in mouse MsrB1 3′-UTR, we analyzed the occurrence and distribution of Form 1 and Form 2 ESTs in dbEST database. 127 mouse ESTs were detected, including 30 Form 2 and 13 Form 1 sequences (other sequences did not cover the region containing alternative sequences). Form 1 was enriched in C57BL/6 mice (6 out of 13), and Form 2 in FVB mice (5/17), mammary gland (5/17) and tumors (7/17). There were two FVB cDNA libraries that contained both forms. Overall, it appears that intron 4 is alternatively spliced in mice in a process that is partially influenced by mouse strain and disease state.

### Characterization of MsrB1 mRNA forms in mice

**Figure 2:**
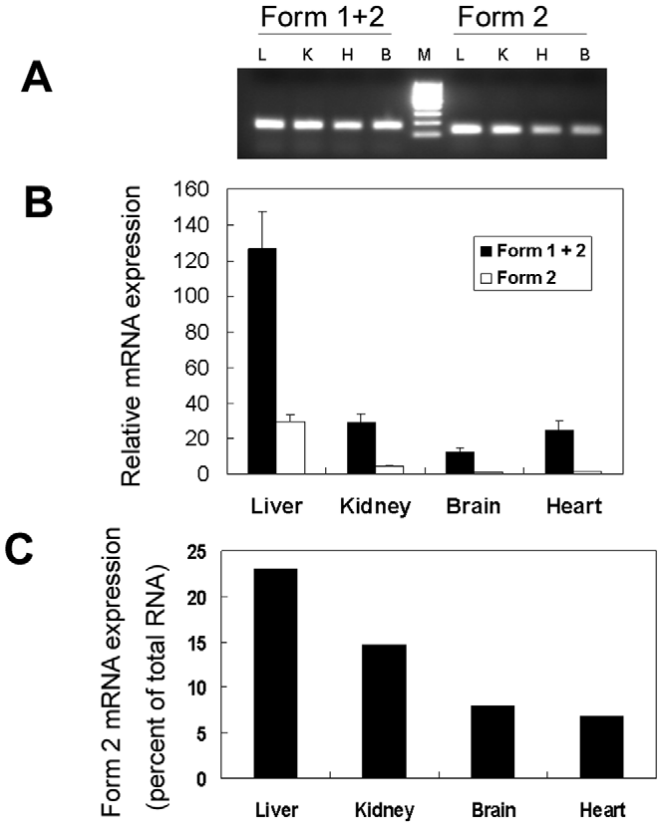
Analysis of MsrB1 mRNA forms in mice. MsrB1 mRNA expression in indicated organs of two C57BL/6 mice was analyzed by real-time PCR. (A) Agarose gel electrophoresis analysis of MsrB1 PCR products. Real-time PCR products derived from mouse MsrB1 mRNA (Forms 1 and 2) and MsrB1 Form 2 mRNA were subjected to 2% agarose gel electrophoresis. L, liver; K, kidney; H, heart; B, brain; M, molecular markers, The size of the band running fastest is 100 bp, and that of the adjacent band is 200 bp. (B) MsrB1 mRNA expression. Expression levels of total MsrB1 mRNA (Forms 1 and 2) and Form 2 mRNA were normalized to the levels of GAPDH mRNA, and the value for Form 2 in the brain was set as 1. The data represent means±SD. (B) Relative expression of MsrB1 Form 2 mRNA. Expression of MsrB1 Form 2 mRNA was normalized to total MsrB1 mRNA.

To directly examine expression of MsrB1 mRNA forms in mice, we analyzed their expression levels in liver, kidney, brain, and heart in two C57BL/6 mice by real-time PCR. Both forms were detected in all mouse tissues examined. Form 2 was 7–25% of the total MsrB1 mRNA (Fig. 2B, C), indicating that Form 1 was the major form. We also amplified the full length MsrB1 cDNA from mouse liver and sequencing revealed Form 1 sequence. Quantitation of MsrB1 mRNA indicated that it ranked in the following order: liver, kidney, heart, and brain. This is also consistent with MsrB1 protein expression in these organs of mice as analyzed by Western blot assays (data not shown).

### Both mRNA forms of mouse MsrB1 support selenoprotein synthesis and efficient Sec insertion

**Figure 3:**
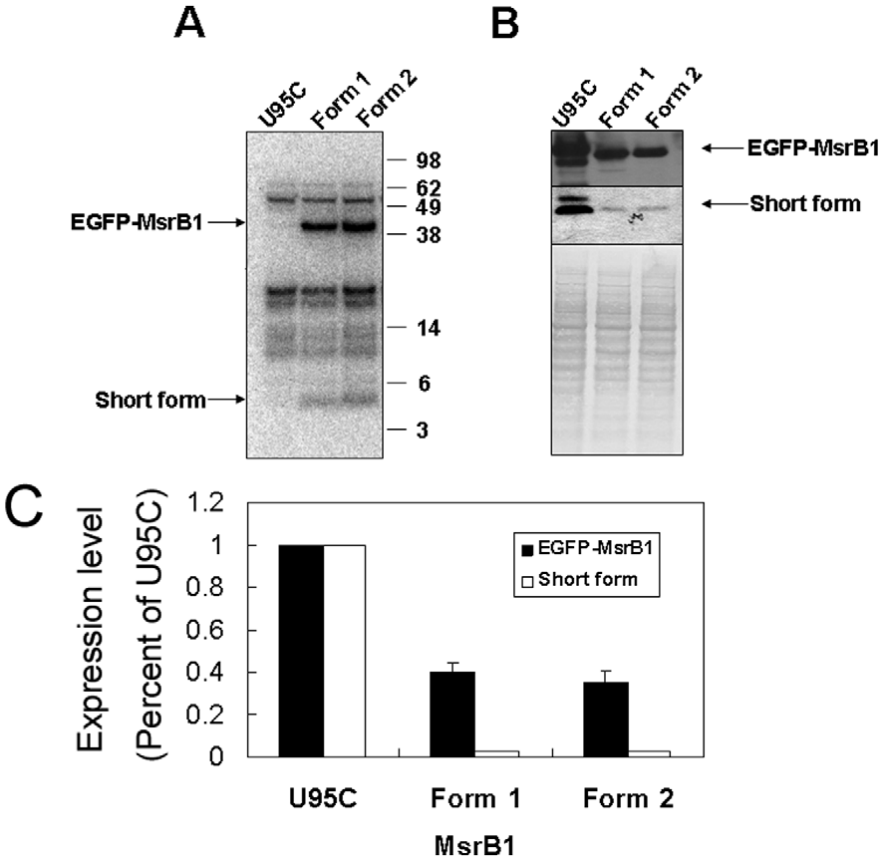
Expression of mouse MsrB1 mRNAs. HEK 293 cells were transfected with the following constructs: pEGFP-MsrB1 Cys mutant (U95C), pEGFP-MsrB1 with Form 1 3′-UTR (Form 1) and pEGFP-MsrB1 with Form 2 3′-UTR (Form 2). 24 h later, the cells were labeled with ^75^Se for an additional 24 h. Protein extracts were prepared and resolved on SDS-PAGE gel, and selenoproteins were visualized with a PhosphorImager (A) or by western blot analysis with MsrB1 antibodies (B, top panel). Coomassie Blue staining is shown as a loading control (B, lower panel). (C) Band intensities corresponding to different forms of MsrB1 (B) were quantified with Image J. Protein expression levels were normalized to that of the U95C form. Arrows indicate migration of a full-length EGFP-MsrB1 (42 kDa) and a short form (5 kDa) of MsrB1.

To test if the two mRNA forms generate functional MsrB1, we prepared the corresponding expression constructs differing in the 3′-UTR and coding for an N-terminal GFP followed by mouse MsrB1 sequences. These constructs were transfected into HEK 293 cells, which were then metabolically labeled with ^75^Se. Both MsB1 mRNA forms generated a full-length GFP-MsrB1, which was labeled with ^75^Se at the same level (Fig. 3A) and generated similar amounts of protein (P>0.05) (Fig. 3B, C). MsrB1 was the major selenoprotein in transfected cells, indicating highly efficient Sec insertion by the MsrB1 SECIS element. Thus, both mRNA forms support protein synthesis and efficient Sec insertion. For comparison, we expressed a Cys mutant of MsrB1 in HEK 293 cells. As expected, it was expressed at higher levels than the Sec-containing form (P<0.01) (Fig 3B, C) (because Sec insertion is slower than Cys insertion), but was not labeled with ^75^Se (Fig. 3A).

### Relationship between mRNA and protein forms of MsrB1

In addition to the two mRNA forms, MsrB1 protein occurs in two protein forms that migrate as 14 and 5 kDa proteins. We tested which of the two mRNA forms gives rise to different protein forms (Fig. 3A) and found that expression of GFP-MsrB1 fusion protein from either mRNA form led to the appearance of a short 5 kDa protein, which was labeled with ^75^Se to the same extent. Thus, each mRNA form can lead to the synthesis of two protein forms.

### Sec is not required to generate the 5 kDa form

MsrB1 is a Sec-containing protein. To test if generation of the short form requires Sec, we examined the production of the 5 kDa form from wild-type MsrB1 and its Sec-to-Cys mutant expressed in HEK 293 cells. The 5 kDa form was detected by western blot analysis with antibodies against recombinant MsrB1 (Fig. 3B) and it was present in cells expressing either Sec-containing or Cys-containing MsrB1 forms. As expected, expression of both full-length and 5 kDa forms was higher in the case of Cys mutant because Sec insertion is slow in mammalian cells (Fig. 3C).

### Roles of amino acids coordinating structural zinc

**Figure 4:**
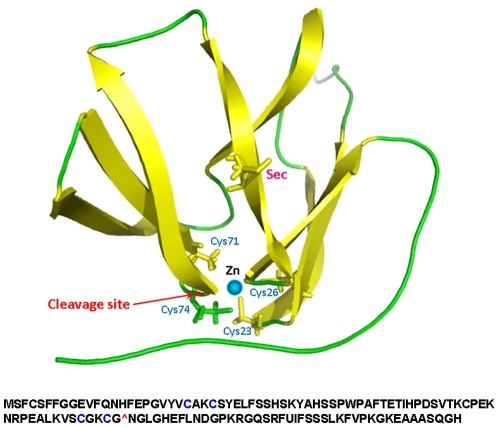
Structural model of mouse MsrB1. The upper panel shows a structural model of mouse MsrB1. Location of four cysteines in two CxxC motifs and of Sec is indicated. The predicted cleavage site is marked with a red dot and is highlighted by an arrow. Lower panel shows mouse MsrB1 sequence. The two CxxC motifs are highlighted in blue. The predicted cleavage site is also shown as “∧”.

**Figure 5:**
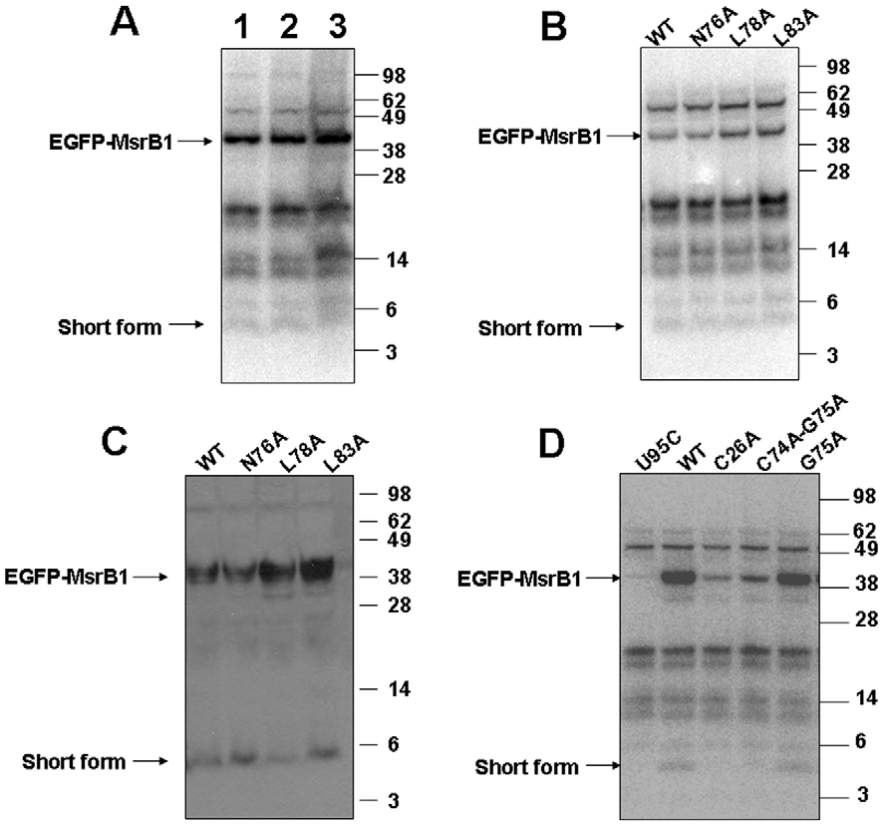
Analysis of the 5 kDa form. (A) Role of sample preparation in the generation of the 5 kDa form. HEK 293 cells were transfected with pEGFP-MsrB1-F1 for 24 h, then labeled with ^75^Se for an additional 24 h. Cells were collected and lysed with sample buffer (Lane 3) or Sigma mammalian cell lysis reagent in the presence (lane 2) or absence (lane 1) of protease inhibitors. Protein extracts were resolved on SDS-PAGE, and selenoproteins were visualized with a PhosphorImager. (B-D) Roles of various amino acids in MsrB1 in generating the 5 kDa form. HEK 293 cells were transfected with pEGFP-MsrB1-F1 or constructs containing mutations in one or two amino acids (as shown above lanes) for 24 h, then labeled with ^75^Se for an additional 24 h. Protein extracts were resolved on SDS-PAGE gels, and selenoproteins were visualized with a PhosphorImager (B, D) or by western blot analysis with MsrB1 antibodies (C). The full-length EGFP-MsrB1 (42 kDa) and the short form (5 kDa) of MsrB1 are shown by arrows.

Immediately upstream of the candidate cleavage site, two Cys residues are located that bind zinc (Fig. 4). These residues form a CxxC motif. There is a second CxxC motif located further upstream that is also involved in zinc coordination [24]. We mutated Cys26 to Ala and also made a mutant that replaced Cys74 and Gly75 with alanines. Each cysteine mutation led to a reduced expression of both 5 and 14 kDa forms (Fig. 5D). As these Cys are required for folding and correct structure of MsrB1, our data implicated protein structure in regulation of MsrB stability and/or maturation to generate the cleaved forms.

In a recent study, we suggested that the 5 kDa form is generated by proteolytic cleavage of the 14 kDa form [13]. Since sample preparation is a common cause of proteolytic cleavage, especially in mammalian cells, we examined the production of the 5 kDa form in HEK 293 cells expressing EGFP-MsrB1, wherein cells were treated or not with complete protease inhibitor during cell lysis or were subjected to direct denaturation by adding SDS-PAGE sample buffer. However, metabolic ^75^Se labeling of samples showed that there was no significant difference in the abundance of the 5 kDa fragment among the samples (Fig. 5A). Thus, the 5 kDa form is not the result of sample preparation.

### The 5 kDa form and alternative translation initiation

Alternative translation initiation is a common mechanism to generate protein isoforms. We examined whether this process is involved in the synthesis of the 5 kDa form. The MsrB1 ORF lacks in-frame ATG codons, which could result in the production of the 5 kDa form, but translation is also known to be initiated in rare cases from other codons, most notable from XTG codons (X is any nucleotide). Two such codons exist in MsrB1: TTG coding for Leu78 and CTG coding for Leu83. We mutated these sites and examined generation of the 5 kDa form. HEK 293 cells expressing either mutant produced the 5 kDa form as detected by PhosphorImager (Fig. 5B,C). These experiments excluded translation initiation as a possible mechanism to generate the 5 kDa form.

### Roles of amino acids in the candidate cleavage region

Mass-spectrometry analyses suggested that the 5 kDa form could result from the cleavage between amino acids Gly75 and Asn76 [13]. To test if these amino acid residues were necessary for generation of the 5 kDa form, we mutated them to alanine residues (Fig. 5B, C, D). Both mutants still produced the short MsrB1 form when expressed in HEK 293 cells.

### The 5 kDa fragment and MsrB1 activity

**Figure 6:**
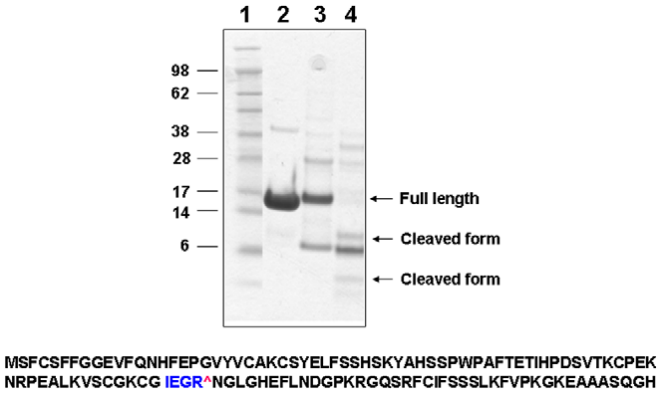
Analysis of recombinant MsrB1. Top panel: Cys mutant of MsrB1 (MsrB1-Cys) (lane 2) and its form with the engineered factor Xa protease cleavage site (MsrB1-Cys-Xa) (lane 3) were analyzed by SDS-PAGE followed by Coomassie Blue staining. MsrB1-Cys-Xa protein was digested with factor Xa and the digestion products (lane 4) were subjected to SDS-PAGE analysis. Molecular weight markers are shown in lane 1. Arrows indicate migration of the uncleaved (full length) protein and cleaved protein fragments (cleaved form). Bottom panel: Sequence of MsrB1-Cys-Xa. The engineered factor Xa site is shown in blue, U95C in orange, and the factor Xa cleavage site is shown by “∧”.

We examined if the cleavage that generates the 5 kDa fragment affects MsrB1 activity. We cloned the predicted N-terminal (amino acids 1–75) and C-terminal (amino acids 76–116) (Cys mutant was used in this experiment because Sec insertion is an inefficient process) segments of MsrB1 into pETDueT vector and expressed them simultaneously in *E. coli*. However, neither fragment was soluble. Since we did not know which protease is involved in the cleavage of MsrB1, we engineered a cleavage site for factor Xa protease by inserting the sequence coding for tetrapeptide IEGR between Gly75 and Asn76 [13]. The resulting recombinant protein was expressed in *E. coli* as His-tagged protein, purified and treated with factor Xa, which cleaved the protein into fragments of expected size (Fig. 6). We found that MsrB1 with the engineered factor Xa cleavage site had some Msr activity, whereas treatment with factor Xa protease *in vitro* resulted in the loss of this activity (Table 1).

**Table 1:** Specific activity of mouse MsrB1-Cys and its mutant forms.

Protein form	Specific activity (nmol/min/mg protein)
MsrB1-Cys	1.5±0.1
MsrB1-Cys-Xa	3.3±0.1
Cleaved MsrB1-Cys-Xa	ND

Catalytic activity of purified recombinant proteins was determined using dabsyl-Met-RO as substrate. MsrB1-Cys: Cys mutant of mouse MsrB1, MsrB1-Cys-Xa: Cys mutant of MsrB1 engineered to contain a factor Xa cleavage site inserted in the coding region between Gly75 an Asn76. Cleaved MsrB1-Cys-Xa: MsrB1-Cys-Xa cleaved with factor Xa. ND: not detected.

### Oxidative stress and the 5 kDa fragment

**Figure 7:**
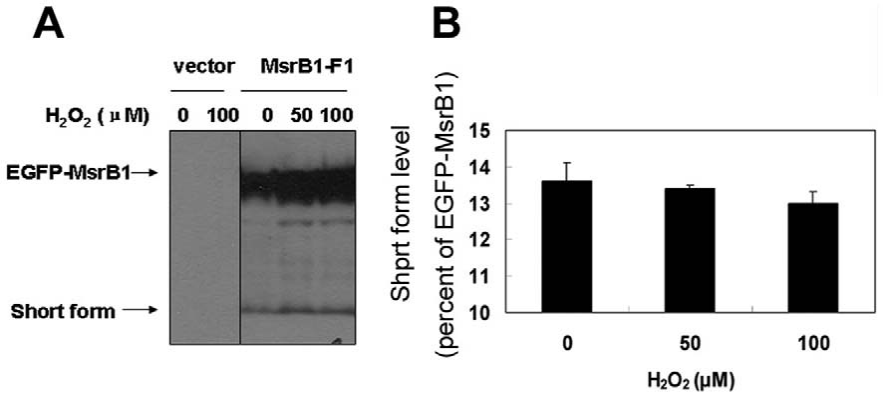
Role of oxidative stress in generating the 5 kDa form. HEK 293 cells were transfected with pEGFP vector alone (vector) or pEGFP-MsrB1-F1 wild-type (WT) for 24 h and then cultured with DMEM containing indicated concentrations of hydrogen peroxide for another 24 h. Protein extracts were resolved on SDS-PAGE and detected by western blot analysis using antibodies specific for MsrB1 (A). MsrB1 band intensities were determined with Image J and MsrB1 short form levels were normalized to those of the EGFP-MsrB1 form (B).

Oxidative stress can lead to oxidation of methionine residues, which are repaired by MsrB1. We examined a possibility that oxidative stress triggers the cleavage of MsrB1, which in turn regulates methionine sulfoxide reduction. HEK 293 cells expressing MsrB1 were treated with hydrogen peroxide and the abundance of the 5 kDa form was examined by Western blot analysis using antibodies specific for MsrB1 (Fig. 7). The data suggested that the production of 5 kDa fragment did not change after the treatment (P>0.05).

### Lack of the 5 kDa fragment in MsrB2

**Figure 8:**
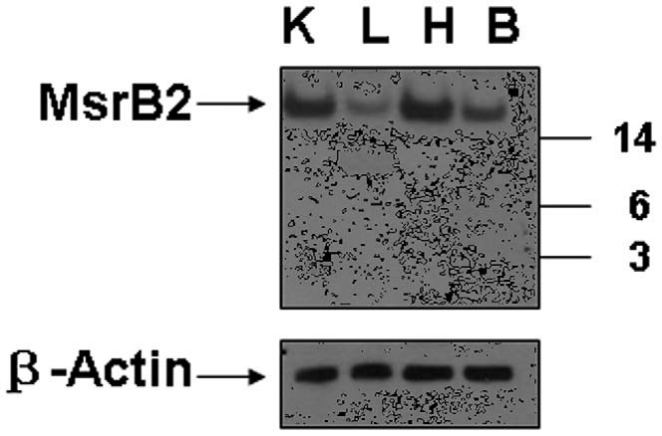
Immunoblot analysis of MsrB2 in mouse tissues. MsrB2 expression was analyzed in C57BL/6 mouse kidney (K), liver (L), heart (H), and brain (B) by western blot using antibodies specific for mouse MsrB2. Migration of MsrB2 is shown by an arrow, molecular weight markers are shown on the right, and the control for protein loading (β-Actin) is also shown.

Finally, we tested whether MsrB homologs may also generate abundant cleavage forms by examining MsrB2 expression and occurrence of full-length and shorter protein forms by immunoblot assays in mouse tissues. Only the full-length form was detected in liver, kidney, heart, and brain (Fig. 8). Thus, an abundant 5 kDa fragment is not a common form of mammalian MsrBs.

## Discussion

This study revealed an unexpected variety of mRNA and protein forms in a small and relatively simple protein, MsrB1. This finding adds a layer of complexity in regulation of this selenoprotein. 3′-UTRs play important roles in mRNA stability, localization, and translation [25]. Eukaryotic selenoprotein mRNAs, including the MsrB1 mRNA, have a stem loop structure, designated SECIS element, located in the 3′-UTR region, which is required for Sec incorporation. A minimal distance between Sec codon and SECIS element is thought to be 51 to 111 nucleotides [26]. An overall mRNA structure is also important for Sec insertion [27]. In this regard, it is surprising that the new form of mouse MsrB1 mRNA lacks a significant part of the 3′-UTR, yet it is as efficient in Sec insertion as the longer mRNA form.

Among 30 mouse MsrB1 ESTs, Form 1 sequences were prevalent in samples from C57BL/6 mice, whereas Form 2 sequences were enriched in FVB mice. In addition, almost half of Form 2 sequences were derived from tumors, whereas no tumors gave rise to ESTs corresponding to Form 1 MsrB1 mRNA. Consistent with the computational analysis, our experiment data suggested that both Form 1 and Form 2 could be detected in C57BL/6 mice, and that Form 1 was the major form. Aberrant splicing is a common feature of cancer cells, and variation in mRNA splicing of certain tumor-related genes has been identified as a biomarker of certain tumors [28].

Computational analysis suggested that, in rats, only one (Form 1) MsrB1 mRNA was detected, whereas all other mammals represented in dbEST possessed Form 2 mRNA. Thus, mouse appeared to be the only mammal well represented in dbEST that showed alternative splicing in MsrB1 mRNA, but it is likely that future studies may find additional mammals with the two mRNA forms.

Alternative splicing is an important mechanism for generating functional and evolutionary diversity of proteins in eukaryotes, and a recent study analyzed occurrence of rodent-specific exons. In that study, separation of genes by the rate of sequence evolution and by gene families has demonstrated that rodent-specific exons are more frequent in rapidly evolving genes and in rodent-specific paralogs [29]. This study is consistent with the idea that that gain of alternative exons is one of the major mechanisms of gene evolution.

Another study examined the possible mechanisms of spliceosomal intron creation in *C. elegans* and defined a process of intronization, i.e., the recruitment of internal exonic sequences in nematodes [30]. It appears that this process is as important as several other mechanisms that lead to intron gain. Intronization was more common than the reverse process, loss of splicing of retained introns, in *C. elegans*. In fact, the process of intronization was proposed to be the major mechanism that drives the evolution of new exons and a major force in the shaping of gene structures [31]. However, a recent study that examined *Cryptococcus* sequences identified only five events of intron creation from internal exonic regions by *de novo* emergence of new splicing boundaries, and no cases of the reverse process, deintronization [32]. Although the extent and mechanisms of intronization in mammals will require further studies, our analysis clearly shows that the evolution of two mRNA forms in mammalian MsrB1 is due to intronization of exonic sequences specifically in a subset of rodents. In this regard, MsrB1 may be a model case of intronization in mammals, where this mechanism has not been yet studied in detail.

Our previous study also revealed two protein forms (14 kDa and 5 kDa) of MsrB1 in mouse tissues and human cell lines. Both forms could be detected by ^75^Se labeling and western blotting with polyclonal MsrB1 antibodies. To provide further evidence that the 5 kDa form corresponds to the C-terminal fragment of MsrB1 containing Sec95, we compared ^75^Se labeling patterns of HEK 293 cells expressing wild-type and Cys mutant of MsrB1. It was clear that the 5 kDa form could only be detected in the case of wild-type MsrB1 (Fig. 3B).

To gain insights into the mechanism of generation of the MsrB1 short form, we examined the correlation between its occurrence and the following possible causes: sample preparation, alternative translation initiation, and Sec presence in the protein. However, none of them correlated with the differential levels of shorter and larger forms. Interestingly, disruption of CxxC motifs that coordinated a structural zinc atom decreased the levels of both full length and short forms. Site-specific proteolysis may be used to both activate and inactivate enzymes [33], [34]. To examine how the cleavage affects MsrB1 activity, we engineered a factor Xa cleavage site at the predicted cleavage site within MsrB1. The recombinant protein exhibited activity, which was lost upon treatment of MsrB1 with factor Xa. While these data may be interpreted in support of protein inactivation by proteolytic cleavage, the artificial nature of the factor Xa site does not allow us to make this conclusion without further verification. We also examined the role of oxidative stress in regulating the expression of the 5 kDa MsrB1 form. Hydrogen peroxide treatment of HEK 293 cells expressing MsrB1 did not affect the abundance of the 5 kDa form. Finally, we tested whether the generation of two protein forms is common to other MsrBs. Only one protein form was detected in the case of MsrB2, a mammalian mitochondrial MsrB. Thus, the production of abundant 5 and 14 kDa forms is not a general feature of mammalian MsrBs.

In summary, we described the process of intronization in a subset of rodents that led to evolution of a new mRNA form of MsrB1. This form was as efficient as the ancestral form in MsrB1 protein synthesis and in Sec insertion. The evolved MsrB1 mRNA form was the major form in C57BL/6 mice. Each of the MsrB1 mRNA forms could generate two protein forms of MsrB1, apparently due to proteolytic cleavage of the enzyme. We examined this process in detail, which excluded several common mechanisms responsible for generation of protein isoforms. However, further studies are needed to determine the specific function of the 5 kDa fragment of MsrB1.
